# Assessment tools for the risk of pressure injury in children: A systematic review

**DOI:** 10.1016/j.ijnsa.2025.100410

**Published:** 2025-08-13

**Authors:** Faustine Dessi, Julien Valeille, Pascale Beloni, Jean Toniolo

**Affiliations:** a CHU Limoges, France; b Inserm U1094, IRD U270, Univ. Limoges, CHU Limoges, EpiMaCT - Epidemiology of chronic diseases in tropical zone, Institute of Epidemiology and Tropical Neurology, OmegaHealth, Limoges, France; c Département Universitaire de Sciences Infirmières, Faculté de Médecine, Université de Limoges, France

**Keywords:** Child, Pediatric, Pressure, Injury, Scale

## Abstract

**Background:**

Pressure injuries in pediatric patients are associated with increased morbidity, mortality, and prolonged hospital stays, making the identification of effective risk assessment tools critical in clinical practice. Accurate risk assessment is essential in clinical practice. Although several assessment tools exist, their applicability and effectiveness vary across pediatric populations.

**Objective:**

To identify and evaluate tools designed to assess the risk of pressure injuries in children, focusing on their characteristics, validation populations, reliability, and applicability across different clinical contexts.

**Design:**

A systematic review was conducted.

**Methods:**

This systematic review (PROSPERO: CRD42024527687) was conducted in accordance with the Cochrane Handbook. Comprehensive searches were performed in five databases—PubMed, Google Scholar, CINAHL, Web of Science, and Embase—for studies published between January 2010 and March 2024. Eligible studies included those describing the development, use, or validation of pressure injury risk assessment tools in children. Two independent reviewers conducted the selection and appraisal process. Methodological quality was assessed using the QUADAS-2 and QAREL checklists, with general reference to COSMIN guidelines.

**Results:**

Of 964 records screened, 28 studies met the inclusion criteria, encompassing research from 15 countries, with Brazil being the most represented. Studies focused on pediatric and neonatal intensive care units, general wards, and surgical units. Ten different risk assessment tools were identified, including the Braden Q, Braden QD, and Glamorgan scales. The Braden QD scale—an adaptation of the Braden Q including medical device-related risk—was the most frequently evaluated. Other tools, such as the Pediatric Pressure Ulcer Prediction and Evaluation Tool and the Braden Q+P, were specifically developed or adapted for pediatric and neonatal use.

**Conclusions:**

All identified tools demonstrated acceptable validity and inter-rater reliability. However, no single tool proved universally applicable across all pediatric contexts. The Braden QD, Braden Q, and Glamorgan scales emerged as the most comprehensive, particularly in intensive care settings, aligning with National and European Pressure Ulcer Advisory Panel recommendations. Further research is warranted to enhance tool accuracy and contextual adaptability across pediatric care environments.

**Guidelines:**

PRISMA.

**What is already known**

• Pressure injuries are a common and serious complication in pediatric hospital settings, with existing risk assessment tools primarily adapted from adult scales.
• The prevalence of pressure injuries in pediatric populations is variable, and current tools often lack specificity for pediatric care contexts.
• There is a knowledge gap in identifying validated and reliable pressure injury risk assessment tools tailored specifically for pediatric populations.

Alt-text: Unlabelled box

**What this paper adds**

• This review identifies ten pressure injury risk assessment scales for pediatric populations, highlighting the Braden QD scale as the most comprehensive tool considering multiple relevant risk factors.
• The paper demonstrates that while several scales are available, their diagnostic accuracy and applicability vary across different pediatric care contexts, underscoring the need for context-specific tool selection.
• The findings emphasize the importance of regularly using validated assessment tools in clinical practice to effectively prevent pressure injuries in pediatric patients.

Alt-text: Unlabelled box

## 1 Background

Pressure injuries are localized damage to the skin and/or underlying soft tissue, typically occurring over a bony prominence or under a medical or other device, as a result of intense and/or prolonged pressure, often in combination with shear. These injuries may present as intact skin or an open ulcer and can be painful. They frequently develop during prolonged hospitalization due to immobility and represent a common concern in both acute and long-term care settings (Bluestein and Javaheri, 2008, Edsberg et al., 2016, Mondragon and Zito, 2025). Among the most significant and increasingly recognized risk factors is the presence of medical devices, which can exert sustained localized pressure on vulnerable anatomical sites (Mondragon and Zito, 2025).

According to various studies, the prevalence of pressure injuries shows significant variation. A systematic review and meta-analysis conducted by Zhang et al. in 2021 (Zhang et al., 2022), based on fifteen studies of prevalence in children and one study involving neonates, found a pooled prevalence of 12.2 %, with a 95 % confidence interval (CI) ranging from [8.0–16.3]. In the same study, a prevalence of 22.8 % was reported in Europe, with a 95 % CI of [3.5–42.2]. A national prevalence survey in France in 2014 recorded a prevalence of 8.1 % (Mondragon and Zito, 2025). However, it is notable that prevalence rates vary significantly between healthcare settings, ranging from 11.8 % in rehabilitation and follow-up care to 5.3 % in obstetric surgery and medicine (Barrois et al., 2017). Verdon et al., in their study conducted in Switzerland, found a prevalence of 4.8 % (n = 17) among children, with rates twice as high in intensive care units (6.06 %; n = 2 out of 33) and neonatal care units (7.06 %; n = 6 out of 85), compared to other pediatric units (3.85 %; n = 9 out of 234) (Verdon et al., 2022).

Pressure injuries are a serious and preventable complication across all hospitalized patient populations (Sterken et al., 2015). The prevention of pressure injuries and the maintenance of skin and tissue integrity are deemed essential objectives within the care process, particularly under the purview of nursing responsibilities. This preventative care for pediatric patients is crucial given the potential implications for quality of life, healthcare costs, and duration of hospital stays (Sterken et al., 2015, Chauvet et al., 2015, Vocci et al., 2023, Galvin and Curley, 2012). Best practice recommendations advocate for the implementation of structured risk assessments to identify at-risk patients and establish preventative methods for pressure injuries (Bluestein and Javaheri, 2008, Galvin and Curley, 2012). In children, the risk of developing pressure injuries can be influenced by several factors, including immobility, lack of mobility, underlying medical conditions, and nutritional status (Lauderbaugh et al., 2021). Accurate assessment of the risk of pressure injuries is vital for implementing appropriate preventative strategies, particularly in neonatal, intensive care, and broader pediatric contexts, especially since there are currently no specific best practice recommendations for pediatrics. Consequently, the need to identify risks to establish preventative measures prompts healthcare providers to utilize numerical risk assessment scales. For adults, there are nearly 40 different risk assessment scales for pressure injuries (Moore and Cowman, 2014). The most commonly used risk assessment tools providing a numeric score include the Modified Norton, Braden, and Waterlow scales (Anthony et al., 2008, Pancorbo-Hidalgo et al., 2006). Each of these tools assigns numerical values to a set of clinical criteria, generating a total risk score that guides clinical decision-making. The Braden scale, validated for adult populations, includes six items—sensory perception, moisture, activity, mobility, nutrition, and friction/shear—each scored from 1 to 4 (from least to most favorable), yielding a total score ranging from 6 to 23. It remains the most widely used pressure injury risk scale in France (Bergstrom et al., 1987, Bergstrom et al., 1987).

To address the specific needs of pediatrics, Quigley and Curley developed the Braden Q scale, which retains the six items of the adult version and adds a seventh on tissue perfusion and oxygenation, and accounts for the child’s psychomotor development (Quigley and Curley, 1996). However, the Braden Q scale solely considers factors related to immobilization and is designed for a limited pediatric age group. In this context, various risk assessment tools for pressure injuries in children have been developed internationally, including the Braden QD scale (Curley et al., 2018). The Braden QD scale, developed by Dr. Matha Curley, is a simplified and revised version of the Braden Q scale and includes risks related to the presence of medical devices (Curley et al., 2018). In addition to these two scales, numerous other tools for assessing the risk of pressure injuries in children exist, such as the Glamorgan scale and the Neonatal Skin Risk Assessment Scale, which are specifically detailed in the systematic review published on the subject in 2013 (Kottner et al., 2013). Among the identified tools, only the Braden, Braden Q, and Braden QD scales have undergone formal validation, primarily in English-speaking populations. Most scales assign a high weighting index to age, rendering them unsuitable for pediatric use. Some scales have been modified or created specifically for use in pediatric care settings (Kottner et al., 2013, Noonan et al., 2011). However, the validity, reliability, and applicability of these tools in specific pediatric contexts vary and are not always clearly defined.

To date, only one systematic literature review published in 2013 by Kottner et al. (Kottner et al., 2013) has examined pressure injury risk assessment scales in children, finding no validated scale in French, thereby hindering the identification of a tool capable of guiding care practices within the French context. Therefore, this systematic review aims to evaluate and synthesize the available evidence on risk assessment tools for pressure injuries in children.

Our research question was: What pressure injury risk assessment tools for children are available in literature?

### 1.1 Main objective

• Identify the various pressure injury risk assessment tools available in children.

### 1.2 Secondary objectives

• Identify the most effective and appropriate tools for predicting pressure injury risk in children.
• Describe the characteristics of tools designed to identify children at risk of developing pressure injuries, including the populations for which they were validated, their intended care settings, age ranges, and health conditions.

## 2 Methods

We conducted a systematic review of the literature based on articles concerning risk assessment tools for pressure injuries in children from January 2010 to March 2024 for data collection. The research protocol for our study was submitted to the International Prospective Register of Systematic Reviews (PROSPERO) under the number CRD42024527687. The checklist "Preferred Reporting Items for Systematic Reviews and Meta-Analyses" (PRISMA) (Page et al., 2021) was adhered to during the writing of this paper. Our methodology was based on the guidelines established by Cochrane (Higgins and Green, 2008) for conducting the literature review and followed the systematic review methods published in 2013 by Kottner et al. (Kottner et al., 2013).

### 2.1 Study type and period

This was a systematic review in which study selection was conducted from 11th March to 26th April 2024, focusing on the tools for evaluating the risk of pressure injuries in children.

### 2.2 Inclusion and exclusion criteria

We included all studies — regardless of design — that addressed the development, validation, or use of pressure injury risk assessment scales in children, published between January 1, 2010, and March 31, 2024, without language restrictions. For this review, “children” were defined according to the legal age of minority applicable in each country where the included studies were conducted. The year 2010 was chosen as it marked the end of the search period for the previous systematic review by Kottner et al. (Curley et al., 2018). Studies solely involving adults were excluded, as were editorials, letters to the editor, case reports, conference abstracts, and any form of literature review.

### 2.3 Research strategy

The search strategy was informed by the recommendations of Bramer et al. (2017) regarding optimal database combinations for systematic reviews. Accordingly, four databases—Embase, Web of Science, CINAHL, and PubMed—were searched, along with Google Scholar to broaden coverage. In line with Bramer et al.'s guidance, only the first ten pages of Google Scholar results were screened to ensure relevance while limiting noise and redundancy (Bramer et al., 2017). The search strategy was developed using the PICOTS (Population Intervention Comparator Outcome Time Study-Design) framework and combined keywords related to the main concepts of the review: (“Pressure Injuries” OR “Pressure Ulcer”) AND (“assessment tools” OR “scales”) AND (“Pediatrics” OR “Children” OR “Infant” OR “Neonates”). Keywords were adapted to the thesaurus and indexing system of each database to ensure optimal coverage. Furthermore, a "title and abstract" search was conducted in PubMed to ensure comprehensiveness. The search strategy was tailored to the specifics of each database, utilizing MeSH terms for PubMed, for example, and employing Boolean operators “AND” and “OR”. The following strategy was used for PubMed: ("Pressure Ulcer"[Mesh] OR "Pressure Injury"[Mesh]) AND ("Child"[Mesh] OR "Pediatrics"[Mesh] OR "Infants"[Mesh] OR "Neonates"[Mesh]) AND ("Risk Assessment"[Mesh] OR "Scales"[Title/Abstract] OR "Evaluation"[Title/Abstract]). The search equations employed for the other databases are available in Appendix 1.

### 2.4 Article selection process

All identified articles were retrieved and exported using the Rayyan QCRI software (Qatar Computing Research Institute, Data Analytics Medical) (Rayyan 2025). Two authors (DF and TJ) conducted an independent review of all articles after removing duplicates. To determine their eligibility, they individually assessed all titles and abstracts, followed by a full-text reading of the articles. Any discrepancies were discussed, and a consensus was reached by both researchers during the two phases of the evaluation. In cases where the two authors could not reach an agreement, another researcher was consulted to resolve the disagreement, and the majority decision was adopted in accordance with the disagreement management charter prepared by the two authors. Any disagreements between reviewers were resolved through discussion and consensus by a third reviewer (BP).

### 2.5 Data extraction and article evaluation

Data extraction was performed independently by two reviewers (DF, TJ) using a customized data extraction table specifically developed for this review and inspired by the structure used in the systematic review by Kottner et al. (Kottner et al., 2013). Prior to full data extraction, a pilot test was conducted on a subset of five studies to ensure consistency, clarity, and alignment between reviewers. Discrepancies were discussed and resolved with the involvement of a third reviewer (BP), who also verified all extracted data (Kottner et al., 2013) Extracted data were : the name of the first author, the year of publication, the country in which the study was conducted, the aim of the study, the tools used to assess the risk of pressure injuries and the score range, the subjects included and their ages, the care units in which the studies were conducted, the method of data collection, the assessors, and the psychometric or metrological properties of the scales. A narrative synthesis was conducted to summarize and compare the characteristics, contexts of use, and psychometric properties of the included assessment tools. To ensure consistency in the evaluation of measurement properties across studies, we systematically applied interpretation thresholds recommended by the COSMIN initiative (Consensus-based Standards for the Selection of Health Measurement Instruments), rather than relying on the criteria proposed by the original study authors. Internal consistency was considered acceptable when Cronbach’s alpha ranged between 0.70 and 0.95. Reliability, including inter-rater and test–retest, was assessed using Intraclass Correlation Coefficient (ICC) values, interpreted as acceptable when ≥0.70, good when ≥0.75, and excellent when ≥0.90. Cohen’s kappa values were considered moderate between 0.41–0.60, substantial between 0.61–0.80, and almost perfect above 0.80. Discriminative ability was evaluated through Area Under the Curve (AUC) values, with thresholds set at ≥0.70 for acceptable, ≥0.80 for good, and ≥0.90 for excellent performance. Correlation coefficients, such as Spearman’s rho, were classified as moderate (0.50–0.69), strong (0.70–0.89), or very strong (≥0.90). For content validity, individual items were judged acceptable when Item-level Content Validity Index (I-CVI) was ≥0.78 and the overall Scale-level Content Validity Index (S-CVI) was ≥0.90. Aiken’s V values ≥0.80 were interpreted as indicating strong expert agreement.

### 2.6 Evaluation of study quality

The methodological quality of the included studies was assessed using the QUADAS-2 (Quality Assessment of Diagnostic Accuracy Studies) and QAREL (Quality Appraisal of Reliability Studies) checklists. These tools were chosen to ensure comparability with the methodology used in the first systematic review on this topic by Kotner et al., which also applied these instruments for quality appraisal (Kottner et al., 2013). They were complemented by a general reference to the COSMIN recommendations (Whiting et al., 2003). All included studies were independently assessed by two reviewers (DF, TJ); all assessments were then reviewed by a third reviewer (BP) to ensure consistency and resolve any discrepancies.

The QUADAS-2 tool was used to evaluate the risk of bias in diagnostic accuracy studies. It includes four domains—patient selection, index test, reference standard, and flow and timing—each rated for risk of bias, with the first three also assessed for concerns regarding applicability (Whiting et al., 2003). Two independent reviewers applied the checklist to each diagnostic study, with disagreements resolved by discussion or, if needed, by a third reviewer.

The QAREL checklist was applied to studies assessing inter- or intra-rater reliability. It consists of 11 items addressing key aspects such as representativeness, blinding, stability of the measured condition, standardization of procedures, and appropriateness of statistical methods (Lucas et al., 2013).

Although we did not formally score the studies using the COSMIN checklist (COSMIN 2024), we referred to its methodological guidance to ensure that the included studies met general standards for study design and reporting of measurement properties, particularly in terms of reliability and measurement error.

Together, the use of QUADAS-2 and QAREL provided a detailed assessment of methodological quality, while COSMIN offered a conceptual framework to guide the overall appraisal process.

## 3 Results

### 3.1 Results findings

**Fig. 1:**
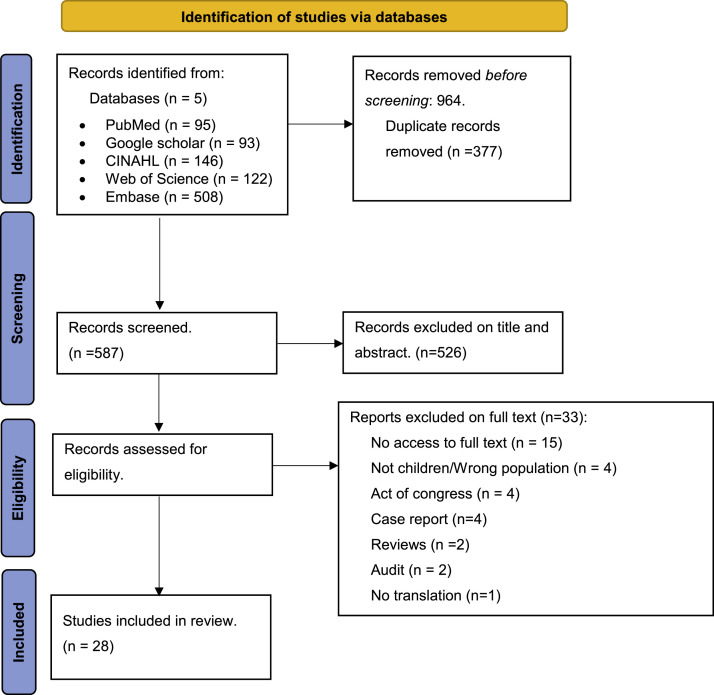
PRISMA Flowchart. From : http://www.prisma-statement.org/.

Our research resulted in a total of 964 articles extracted from the five databases. After removing duplicates, we excluded 526 articles after reading the title and abstract. Finally, after reading the full text of the 61 articles deemed relevant, only 28 were included in the analysis. Fig. 1 presents the PRISMA flow diagram describing the selection process (Fig. 1).

### 3.2 Characteristics of included studies

The included studies focused on patients from a total of 15 countries, with the majority being conducted in Brazil (n = 8). All 28 included studies were conducted in hospital settings: in pediatric (n = 15) and neonatal (n = 10) intensive care units, with a few rare studies conducted in general medicine (n = 1) and surgery (n = 2) departments. The overall age range of patients included in the studies was between 0 and 21 years, with some studies including newborns and premature infants. The scales used to assess pressure injury risk in the pediatric population included the Braden Q scales (Maia et al., 2011, Tume et al., 2014, De Lima et al., 2016, Vocci et al., 2017, Vocci et al., 2020, de Mendonça Carvalho et al., 2015), the Braden QD scales (Verdon et al., 2022, Curley et al., 2018, de Mendonça Carvalho et al., 2015, Puspitasari et al., 2020), and the Glamorgan scale (Vidal Santos et al., 2022, Kottner et al., 2014, Willock et al., 2008). David et al developed a new scale in 2014, the Pediatric Pressure Ulcer Prediction and Evaluation Tool (Sterken et al., 2015). This scale was used in the United States to assess pressure injury risk in the pediatric population admitted to neonatal intensive care units. It was designed to assess pressure injury risk in newborns up to 18 years of age. In 2012, Galvin et al modified the Braden Q scale to Braden Q+P to assess pressure injury risk in the surgery department at Boston Children's Hospital (Galvin and Curley, 2012). This tool was designed to be used in surgical departments and operating rooms to prevent pressure injuries in newborns and patients up to 31 years old undergoing surgical procedures. The detailed characteristics of the included studies in the review are summarized in Table 1.

**Table 1:** Characteristics of included studies.

Author & Publishing year	Survey country	Scale name & score range	Subject’s age	Sample size	Care unit	Psychometric properties
	**EUROPE**					
(Kottner et al., 2014)	Germany	Glamorgan: 0-42	-	30	Pediatric cardiac unit	Agreement level = 48 %, ICC = 0.34 5(95 % CI 0.12–0.57), r = 0.68
(Curcio et al., 2022)	Italy	Neonatal Skin Risk Assessment Scale: 6-24	0-28days	-	Neonatal Intensive Care Unit	Content Validity Index (CVI)= 0.92; Aiken’s coefficient = 0.85-0.97
(Curcio et al., 2024)	Italy	i- Neonatal Skin Risk Assessment Scale: 6-24	0-28days	200	Neonatal Intensive Care Unit	α Cronbach= 0.86; McDonalds omega coefficient= 0.86 ICC: intra observer reliability= 0.99; inter observer reliability= 0.98
(Verdon et al., 2022)	Switzerland	Braden QD: 1-23	Premature infants up to 21 years of age	352	Medicine; Surgery; intensive care unit, neonatology	α Cronbach= 0.710
(Willock, 2013)	United Kingdom	Glamorgan: 0-42	1days-17years	27	All pediatric care unit	Cohen’s kappa: k = 0,867 (overt data), k = 0,763 (covert data); spearman’s rho: r = 0,976 (overt data), r = 0,727 (covert data)
(Tume et al., 2014)	United Kingdom	Braden Q: 7-28	0-14years	891	Pediatric Intensive Care Unit	Area under the curve: AUC = 0.87(95 % CI: 0.75–0.98)
	**AMERICA**					
(Maia et al., 2011)	Brazil	Braden Q: 7-28	1month-13years	35	Intensive Care Unit	Cronbach coefficient (α = 0.936); Spearman (r=0.9949); ICC = 0.995 (intra-rater), ICC = 0.998 (inter-rater)
(de Mendonça Carvalho et al., 2015)	Brazil	Braden Q: 7-28	29days-11years	121	Pediatric Intensive Care Unit	Cronbach α = 0.915; ICC = 0.82; AUC = 0.77
(De Lima et al., 2016)	Brazil	Braden Q: 7-28	0-28days, Preterm infants > weeks’ gestational age	50	Neonatal Intensive Care Unit	Pearson’s coefficient r = 0.96 (N/I Braden and Braden Q) inter ratter reliability: r = 0.99, ICC = 0.986 intra ratter reliability: r = 0.87, ICC = 0.791
(Vocci et al., 2017)	Brazil	Braden Q: 7-28	30days-18years	21	Pediatric Intensive Care Unit	Pearson’s correlation: r = 0.2477 (hospitalization time and Braden Q’s score)
(Vocci et al., 2020)	Brazil	Braden Q: 7-28	0-15years	34	Pediatric Intensive Care Unit	Not available data
	**AMERICA**					
(Vocci et al., 2021)	Brazil	Glamorgan : 0-42	0-18years	-	Pediatric Intensive Care Unit	Not available data
(Vidal Santos et al., 2022)	Brazil	Braden QD: 0-20	<24hours	105	Intensive care unit	α Cronbach = 0.773; Kappa coefficient = 0.688; CVI≥0,80
(Vocci et al., 2023)	Brazil	Glamorgan: 0-42	30days-15years	83	Pediatric Intensive Care Unit	Glamorgan: α Cronbach = 0.3; AUC = 0.77; Braden Q: α Cronbach = 0.7; AUC = 0.78
(Lauderbaugh et al., 2021)	San Diego	Braden Q and Braden QD: No risk-at risk	1month-21years	45	All care areas	Mcnemar (comparison of Braden Q and Braden QD)
(Galvin and Curley, 2012)	United state	Braden Q+P: Yes/No	Newborn-31years	-	Surgical services	Not available data
(Sterken et al., 2015)	United state	Pediatric Pressure Ulcer Prediction and Evaluation Tool: 9-26	Newborn-18years	108	Neonatal Intensive Care Unit	Inter-rater reliability: k = 0.718; agreement level: k = 0.349 (Pediatric Pressure Ulcer Prediction and Evaluation Tool and Braden or Braden Q), k = 0,044 (Pediatric Pressure Ulcer Prediction and Evaluation Tool, Glamorgan)
(Foster et al., 2017)	United state	Skin Injury Risk Assessment and Prevention scale: At Risk-No at risk	<30days, 31days-17years, 18years and older	385	All patient from all inpatient units	Pearson’s correlation coefficient: r = 0.725 (Skin Injury Risk Assessment and Prevention scale and Neonatal Skin Risk Assessment Scale), r = −0.634 (Skin Injury Risk Assessment and Prevention scale and Braden Q), r = −0.778 (Skin Injury Risk Assessment and Prevention scale and Braden); Cronbach coefficient: α = 0.556; ICC = 0.878 (inter ratter reliability)
(Alderden et al., 2017)	United state	Braden: 6-23	Younger than 18 years, adults	6377	Intensive care unit, level 1 trauma center	Cox regression
(Curley et al., 2018)	United state	Braden QD: 0-20	Preterm to 21 years	625	All inpatients unit except psychiatric unit	AUC = 0.72, 95 % CI = [0.67-0.78]
(Riccioni et al., 2019)	Texas	Braden, Braden Q: 9-18	21days - 8years	115	Pediatric Intensive Care Unit, Cardiac Intensive Care Unit, Progressive Care Unit, Neurology unit, rehabilitation, and epilepsy monitoring unit	Braden: ICC = 0.894, 95 % CI = [0.823–0.938]; Weighted Kappa coefficients = 0.415-0.921 Braden Q: ICC = 0.7266, 95 % CI = [0.585–0.824]; Weighted Kappa coefficients = 0.429-0.699
**ASIA**						
(Luo et al., 2021)	China	Waterlow: 10-20; Braden Q: 7-28; Glamorgan: 0-42	28days-14years	342	Pediatric Intensive Care Unit; general pediatric ward	AUC in PICU: Waterlow (0.883), Braden Q (0.733), Glamorgan (0.800); AUC in general ward: Waterlow (0.870), Braden Q (0.924), Glamorgan (0.923)
Shi and Li, (2023)	China	Chinese version of the Braden QD scale: 0-20; Chinese version of the N/I Braden Q scale: 8-32	Preterm-28days	410	Neonatal Intensive Care Unit	N/I Braden Q: α Cronbach= 0.806, AUC = 0.879, 95 % CI = [0.843–0.909] Braden QD: α Cronbach = 0.727, AUC = 0.857, 95 % CI = [0.813–0.902]
(Puspitasari et al., 2020)	Indonesia	Braden QD	1month-18years	61	Pediatric Intensive Care Unit	α Cronbach >0.6; Braden: r = 0.532-0.833. Braden Q = 0.528-0.804
(Yalçın Baltacı et al., 2020)	Turkey	NBQBRD: 8-32	0-28days	114	Neonatal Intensive Care Unit	α Cronbach = 0.896; correlation coefficient = 0.88 (validity)
(Sari and Altay, 2017)	Turkey	Neonatal Skin Risk Assessment Scale : 6-24	0-28days	130	Neonatal Intensive Care Unit	α Cronbach = 0.88 (Reliability coefficient), AUC = 0.79, KMO test = 0.73
**OCEANIA**						
(Broom et al., 2017)	Australia	Skin Risk Assessment and Management Tool: 8-32	Neonates born between 24-44 week’s gestation	90	Neonatal Intensive Care Unit	Not available data
(Willock et al., 2016)	Jordan, Australia	Braden Q: 7-28, Glamorgan: 0-42	<18years	513	Pediatric Intensive Care Unit, Neonatal Intensive Care Unit, General Internal Medicine Unit	Glamorgan score AUC = 0.748, 95 % CI = [0.673-0.822] Braden Q score : AUC = 0.820, 95 % CI = [0.760-0.880]

### 3.3 Scales for assessing pressure injury risk in children

In total, 10 pressure injuries risk assessment scales were used in the 28 included studies. Fourteen out of the 28 studies included in the review described standardized pressure injury risk assessment scales for children. Two new scales were developed for neonatal intensive care units (Sterken et al., 2015, Yalçın Baltacı et al., 2020), and another was developed for all hospital units (Lauderbaugh et al., 2021) . Galvin et al made modifications to the Braden Q scale by adding preventive interventions to develop the Braden Q+P scale. The "activity" subscale of the Braden Q scale was omitted in the new Braden Q+P scale since all postoperative patients are under anesthesia and thus immobilized, and the mobility subscale was eliminated, translated, and integrated with the duration of the procedure to determine the effect of lengthy interventions on the development of pressure injuries (Galvin and Curley, 2012). Pediatric patients, infants, newborns, and preterm infants are the target population for other scales. Some authors, Maia et al (Maia et al., 2011), Vocci et al (Vocci et al., 2017), translated the Braden Q scale into their native language of their country and adapted them to their culture without modifying the items of the scale. Similarly, the Glamorgan scale, the Neonatal Skin Risk Assessment Scale, the Neonatal Braden Q, and the Braden QD were translated and culturally adapted in various countries such as Brazil, Turkey, Switzerland, and China (Vocci et al., 2023, Maia et al., 2011, De Lima et al., 2016, Santos and Cravo, 2016, Vocci et al., 2021, Willock et al., 2016, Foster et al., 2017, Shi and Li, 2023). However, these authors kept the same items from the source scale and obtained permission from the original authors before proceeding with the translation of the scales. In terms of the content evaluated by the scales, as shown in Table 2, there are significant differences between the elements assessed by the various scales. The "mobility" item was part of all scales (Verdon et al., 2022, Sterken et al., 2015, Galvin and Curley, 2012, Bergstrom et al., 1987, Vocci et al., 2017, Kottner et al., 2014, Willock et al., 2016, Shi and Li, 2023, Broom et al., 2017, Curcio et al., 2024), followed by nutrition (included in 9 scales), activity (included in 9 scales), sensory perception (included in 8 scales), and friction or shear as well as tissue perfusion or oxygenation (included in 7 scales). However, it is noted that the "moisture" item was only included in 3 scales (Galvin and Curley, 2012, Vocci et al., 2017, Shi and Li, 2023).

**Table 2:** Suggested items for pediatric pressure ulcer risk assessment scales.

Items	Braden (Alderden et al., 2017, Riccioni et al., 2019)	Braden Q (Lauderbaugh et al., 2021, Maia et al., 2011, Tume et al., 2014, De Lima et al., 2016, Vocci et al., 2017, Vocci et al., 2020, de Mendonça Carvalho et al., 2015, Willock et al., 2016, Riccioni et al., 2019, Luo et al., 2021)	Braden QD (Verdon et al., 2022, Lauderbaugh et al., 2021, Curley et al., 2018, Puspitasari et al., 2020, Vidal Santos et al., 2022, Shi and Li, 2023)	Braden Q+P (Galvin and Curley, 2012)	NBQBRD (Yalçın Baltacı et al., 2020)	Skin Risk Assessment and Management Tool (Broom et al., 2017)	Glamorgan (Vocci et al., 2023, Kottner et al., 2014, Vocci et al., 2021, Willock et al., 2016, Luo et al., 2021, Willock, 2013)	Pediatric Pressure Ulcer Prediction and Evaluation Tool (Sterken et al., 2015)	Skin Injury Risk Assessment and Prevention scale (Foster et al., 2017)	Neonatal Skin Risk Assessment Scale (Curcio et al., 2024, Curcio et al., 2022, Sari and Altay, 2017)
Mobility	x	x	x	x	x	x	x	x	x	x
Nutrition	x	x	x	x	x	x	x	x		x
Continence/Incontinence							x			
Weight according to age							x			
Skin condition/skin damage				x				x		
Medical devices				x			x	x	x	
Moisture	x					x		x		x
Tissue perfusion/oxygenation		x	x	x	x		x	x	x	
Age gestational					x	x			x	
Sensory perception	x	x	x	x	x	x		x	x	
Respiratory support						x				
Skin integrity						x				
Blood collection						x				
Other position than decubitus				x						
Humidity		x		x	x					
Activity	x	x	x	x	x	x		x	x	x
Mental state										x
Number of medical devices			x							
Repositionability/Skin prote			x							
General physic condition										x
Pyrexia							x			
Serum albumin <35 g/l							x			
Anemia							x			
Friction/shear	x	x	x	x	x			x	x	
Total	6	7	8	10	8	9	9	9	7	6

### 3.4 Mobility/Activity

Mobility is the common factor across all assessment scales (Verdon et al., 2022, Sterken et al., 2015, Galvin and Curley, 2012, Bergstrom et al., 1987, Vocci et al., 2017, Kottner et al., 2014, Willock et al., 2016, Shi and Li, 2023, Broom et al., 2017, Curcio et al., 2024). The Braden Scale and its derivatives (Braden QD, Braden Q) use four-level Likert scales ranging from: 1-complete immobility to no 4-limitations (Verdon et al., 2022, Lauderbaugh et al., 2021, Curley et al., 2018, Maia et al., 2011, Tume et al., 2014, De Lima et al., 2016, Vocci et al., 2017, Vocci et al., 2020, de Mendonça Carvalho et al., 2015, Puspitasari et al., 2020, Vidal Santos et al., 2022, Willock et al., 2016, Shi and Li, 2023, Alderden et al., 2017, Riccioni et al., 2019, Luo et al., 2021). Others tools, such as the Pediatric Pressure Ulcer Prediction and Evaluation Tool (Sterken et al., 2015) or the Neonatal/Infant Braden Q (Yalçın Baltacı et al., 2020), adapt this assessment to developmental motor abilities. They consider, for example, the ability to be held in a sitting position, to turn the head, or to move limbs spontaneously. Physical activity is often included within the mobility dimension, except in the classic versions of the Braden Scale, where it is treated as a separate item.

### 3.5 Sensory perception

Sensory perception is consistently assessed across all ten scales. In adults, it is linked to the level of consciousness and the ability to report discomfort. In pediatrics, tools such as the Skin Injury Risk Assessment and Prevention scale (Foster et al., 2017) or the Neonatal Braden Q (Yalçın Baltacı et al., 2020) consider behavioural responses to stimuli, including the ability to self-soothe or to respond to touch, light, and sound. The i-Neonatal Skin Risk Assessment Scale (Curcio et al., 2024) also emphasizes the impact of sedation and drug-induced paralysis on sensory perception.

### 3.6 Skin moisture

The level of exposure to moisture is a universal factor across all assessment scales. It is most often rated from “constantly moist” to “rarely moist,” with neonatal scales providing more detailed criteria such as the frequency of diaper or bedding changes. The Braden QD (Verdon et al., 2022, Lauderbaugh et al., 2021, Curley et al., 2018, Puspitasari et al., 2020, Vidal Santos et al., 2022, Shi and Li, 2023) and the Pediatric Pressure Ulcer Prediction and Evaluation Tool (Sterken et al., 2015) scales highlight the links between moisture and urinary or fecal incontinence, while the Skin Injury Risk Assessment and Prevention scale (Foster et al., 2017) specifies the types of secretions involved (drainage, perspiration, stomas).

### 3.7 Friction/Shear

Friction is assessed in nearly all seven scales (Verdon et al., 2022, Sterken et al., 2015, Galvin and Curley, 2012, Lauderbaugh et al., 2021, Curley et al., 2018, Maia et al., 2011, Tume et al., 2014, De Lima et al., 2016, Vocci et al., 2017, Vocci et al., 2020, de Mendonça Carvalho et al., 2015, Puspitasari et al., 2020, Vidal Santos et al., 2022, Yalçın Baltacı et al., 2020, Willock et al., 2016, Foster et al., 2017, Shi and Li, 2023, Alderden et al., 2017, Riccioni et al., 2019, Luo et al., 2021), except for the oldest ones. It is defined as the movement of the skin against the support surface, while shear refers to the displacement of internal structures. Scales such as the Braden QD (Verdon et al., 2022, Lauderbaugh et al., 2021, Curley et al., 2018, Puspitasari et al., 2020, Vidal Santos et al., 2022, Shi and Li, 2023), the Skin Injury Risk Assessment and Prevention scale (Foster et al., 2017), and the Pediatric Pressure Ulcer Prediction and Evaluation Tool (Sterken et al., 2015) provide details on potential causes (e.g., contractures, restless behaviours, spasticity) as well as repositioning strategies. The Neonatal/Infant Braden Q (Yalçın Baltacı et al., 2020) also evaluates friction resulting from agitation or sliding down in bed.

### 3.8 Nutrition

All scales take nutritional status into account, with a gradation ranging from: 1-exclusive parenteral nutrition to 4-full oral feeding, except for the Braden QD (Verdon et al., 2022, Lauderbaugh et al., 2021, Curley et al., 2018, Puspitasari et al., 2020, Vidal Santos et al., 2022, Shi and Li, 2023) scale, which uses a 0- Adequate age-appropriate intake of calories/Protein to support metabolism and growth to 2-Inadequate age-appropriate intake of calories and protein to support metabolism and growth scoring system. Others scales, such as the i-Neonatal Skin Risk Assessment Scale (Curcio et al., 2024) and the Braden Q (Lauderbaugh et al., 2021, Maia et al., 2011, Tume et al., 2014, De Lima et al., 2016, Vocci et al., 2017, Vocci et al., 2020, de Mendonça Carvalho et al., 2015, Willock et al., 2016, Riccioni et al., 2019, Luo et al., 2021), consider both the route of administration and the ability to maintain an appropriate weight. The Neonatal Braden Q (Yalçın Baltacı et al., 2020) even specifies daily caloric intake and weight thresholds to assess nutritional adequacy.

### 3.9 Tissue perfusion/oxygenation

This factor, which was absent from the earlier versions of the Braden scale (Alderden et al., 2017, Riccioni et al., 2019), has now become central in recent assessment tools. The Australian scale, Braden QD, the Skin Injury Risk Assessment and Prevention scale (Foster et al., 2017), and the Neonatal Braden Q (Yalcin et al., 2018) evaluate oxygenation, hemodynamic stability, oxygen saturation, and capillary refill time. These physiological parameters provide a more accurate estimation of ischemic injury risk in the most vulnerable patients.

### 3.10 Medical devices and skin integrity

External medical devices (such as probes, catheters, and masks) are incorporated into recent pediatric scales with specific weighting based on their number and location. Some scales, like the Skin Injury Risk Assessment and Prevention scale (Foster et al., 2017), also evaluate whether the skin beneath the device is protected. Additionally, scales such as the Skin Injury Risk Assessment and Prevention scale (Foster et al., 2017) and the Pediatric Pressure Ulcer Prediction and Evaluation Tool (Sterken et al., 2015) include a direct assessment of the skin condition (redness, wounds, loss of integrity).

### 3.11 Gestational age

The Neonatal Braden Q, the Skin Injury Risk Assessment and Prevention scale, and the Skin Risk Assessment and Management Tool were specific to neonatal scales (Yalçın Baltacı et al., 2020, Foster et al., 2017, Broom et al., 2017), gestational age is a key factor reflecting physiological and skin maturity. These scales categorize newborns into groups (<28 weeks, 28–33 weeks, etc.), assigning higher risk scores to extremely premature infants.

### 3.12 Evaluation of the quality of included studies

#### 3.12.1 Diagnostic accuracy

Most of the included studies use a prospective study design to evaluate the risk of pressure injuries in children. Authors who culturally adapted scales in their native language followed a translation methodology and conducted a pilot study in the pediatric population based on the context of use of the original scale. Most studies met nine out of the eleven quality criteria of QUADAS-2 checklist. However, two studies met eight (Vidal Santos et al., 2022, Foster et al., 2017) and six (Galvin and Curley, 2012, Curcio et al., 2024) out of the eleven quality criteria of QUADAS-2 checklist (Table 3). Since preventive measures were not considered in most studies, it is likely that the estimation of validity may be biased. On the other hand, four authors did not provide data on the methodological quality of validity (Galvin and Curley, 2012, Vocci et al., 2020, Vocci et al., 2021, Curcio et al., 2024).

**Table 3:** Quality assessment according to QUADAS.

Items	Maia et al (2011)	Galvin and Curley (2012)	Willock et al (2013)	Sterken et al. (2015)	Tume et al (2014)	Kottner et al (2014)	de Mendonça Carvalho et al. (2015)	de Lima et al (2016)	Willock et al (2016)	
1-Was a consecutive or random sample of patients enrolled?	Y	Y	Y	Y	Y	Y	Y	Y	Y	
2- Was a case-control design avoided?	Y	Y	Y	Y	Y	Y	Y	Y	Y	
3-Did the study avoid inappropriate exclusions?	Y	Y	Y	Y	N	Y	Y	Y	Y	
4- Were the index test results interpreted without knowledge of the result of the reference standard?	N	U	N	U	Y	N	U	N	N	
5- If a threshold was used, was it pre-specified?	Y	U	Y	Y	Y	Y	Y	Y	Y	
6- Is the reference standard likely to correctly classify the target condition?	Y	Y	Y	Y	Y	Y	Y	Y	Y	
7- Were the reference standard results interpreted without knowledge of the results of the index test?	N	N	U	U	U	U	N	U	N	
8- Was there an appropriate interval between index test(s) and reference standard?	Y	U	Y	Y	Y	Y	Y	Y	Y	
9- Did all patients receive a reference standard?	Y	Y	Y	Y	Y	Y	Y	Y	Y	
10- Did all patients receive the same reference standard?	Y	Y	Y	Y	Y	Y	Y	Y	Y	
11- Were all patients included in the analysis?	Y	U	Y	Y	Y	Y	Y	Y	Y	
Y= Yes, N= No, U= Unclear										
Items	Foster et al (2017)	Alderden et al (2017)	Vocci et al (2017)	Sari & Altay(2017)	Broom et al (2017)	Curley et al (2018)	Riccioni et al (2019)	Vocci et al (2020)	Yalçin et al (2020)	
1-Was a consecutive or random sample of patients enrolled?	Y	Y	Y	Y	Y	Y	Y	Y	Y	
2- Was a case-control design avoided?	Y	Y	Y	Y	Y	Y	Y	Y	Y	
3-Did the study avoid inappropriate exclusions?	Y	Y	Y	Y	Y	Y	Y	Y	Y	
4- Were the index test results interpreted without knowledge of the result of the reference standard?	N	U	N	N	U	U	N	N	N	
5- If a threshold was used, was it pre-specified?	Y	Y	Y	Y	U	Y	Y	U	Y	
6- Is the reference standard likely to correctly classify the target condition?	Y	Y	Y	Y	Y	Y	Y	Y	Y	
7- Were the reference standard results interpreted without knowledge of the results of the index test?	N	U	U	U	U	U	N	U	N	
8- Was there an appropriate interval between index test(s) and reference standard?	Y	Y	Y	Y	U	Y	Y	U	Y	
9- Did all patients receive a reference standard?	Y	Y	Y	Y	Y	Y	Y	U	Y	
10- Did all patients receive the same reference standard?	Y	Y	Y	Y	Y	Y	Y	U	Y	
11- Were all patients included in the analysis?	Y	Y	Y	Y	U	Y	Y	U	Y	
Y= Yes, N= No, U= Unclear										
Items	Puspitasari et al (2020)	Vocci et al (2021)	Lauderbaug et al (2021)	Luo et al (2021)	Vidal Santos et al (2022)	Verdon et al (2022)	Curcio et al (2022)	Vocci et al (2023)	Shi and Li (2023)	Curcio et al (2024)
										
1-Was a consecutive or random sample of patients enrolled?	Y	Y	Y	Y	Y	Y	Y	Y	Y	Y
2- Was a case-control design avoided?	Y	Y	Y	Y	Y	Y	Y	Y	Y	Y
3-Did the study avoid inappropriate exclusions?	Y	Y	Y	Y	Y	Y	Y	Y	Y	Y
4- Were the index test results interpreted without knowledge of the result of the reference standard?	N	U	N	N	N	U	N	N	N	U
5- If a threshold was used, was it pre-specified?	Y	U	Y	Y	Y	Y	Y	Y	Y	Y
6- Is the reference standard likely to correctly classify the target condition?	Y	U	Y	Y	Y	Y	Y	Y	Y	Y
7- Were the reference standard results interpreted without knowledge of the results of the index test?	U	Y	N	N	N	U	N	N	N	U
8- Was there an appropriate interval between index test(s) and reference standard?	Y	U	Y	Y	Y	Y	Y	Y	Y	U
9- Did all patients receive a reference standard?	Y	U	Y	Y	Y	Y	Y	Y	Y	Y
10- Did all patients receive the same reference standard?	Y	Y	Y	Y	U	Y	Y	Y	Y	Y
11- Were all patients included in the analysis?	Y	U	Y	Y	Y	Y	Y	Y	Y	Y

Y= Yes, N= No, U= Unclear

The AUC, ICC, CVI, and percentage of agreement were calculated to demonstrate the content validity of the different scales used to assess the risk of pressure injuries in children. In studies where they were evaluated, the calculations demonstrated that the scales used are valid and can be used to measure the risk of pressure injuries in children. The results of the methodological quality evaluation are presented in Table 3.

#### 3.12.2 Reliability and agreement

The reliability and inter-rater and intra-rater agreement were assessed in accordance with the recommendations of COSMIN (COSMIN 2024). Twenty-four studies provided data on reliability and agreement among raters out of the 28 studies included in our review. In all studies, the authors used representative samples of raters and subjects. The raters were not informed of their respective scores, and the time interval between evaluations was considered appropriate. Four authors did not provide data on the reliability and agreement of concordance among raters. Agreement proportions, Kappa coefficient, Cronbach's alpha, ICC, and area under the curve were calculated in most studies to determine the inter-rater validity of the scales. (Vocci et al., 2017;Puspitasari et al., 2020) used Pearson r coefficients which were not indicated to indicate reliability. Excellent inter- and intra-rater reliability, good consistency was observed on all the scales used in the 24 studies.

All these psychometric properties of the different scales show that these scales are valid and reliable for measuring the risk of pressure injuries in the pediatric and neonatal population. The scales identified in this review have good psychometric properties and vary in the age groups for use and in the items evaluated to arrive at the risk of pressure injuries. The results of the methodological quality assessment on reliability are presented in the table available in Table 4.

**Table 4:** Quality assessment according to QUAREL.

Items	Maia et al (2011)	Galvin & Curley (2012)	Willock et al (2013)	Sterken et al (2015)	Tume et al (2014)	Knotter et al (2014)	de Mendonça Carvalho et al (2015)	de Lima et al (2016)
1- Was the test evaluated in a sample of subjects who were representative of those to whom the authors intended the results to be applied ?	Y	Y	Y	Y	Y	Y	Y	Y
2- Was the test performed by raters who were representative of those to whom the authors intended the results to be applied ?	Y	Y	Y	Y	Y	Y	Y	Y
3- Were raters blinded to the findings of other raters during the study ?	Y	N	Y	Y	U	U	N	Y
4- Were raters blinded to their own prior findings of the test under evaluation?	NA	N	U	U	NA	N	U	U
5- Were raters blinded to the subjects' disease status or the results of the accepted reference standard for the target disorder (or variable) being evaluated ?	NA	N	N	NA	N	N	U	N
6- Were raters blinded to clinical information that was not intended to form part of the study design or testing procedure ?	Y	N	U	U	U	U	U	U
7- Were raters blinded to additional cues that are not part of the test ?	U	N	U	U	U	U	U	U
8- Was the order of examination varied ?	Y	U	Y	Y	Y	N	U	Y
9- Was the stability (or theoretical stability) of the variable being measured considered when determining the suitability of the time interval among repeated measures ?	U	Y	Y	Y	Y	U	U	Y
10- Was the test applied correctly and interpreted appropriately ?	Y	Y	Y	Y	Y	Y	Y	Y
11- Were appropriate statistical measures of agreement used ?	Y	N	Y	Y	N	Y	N	Y
Y= Yes, N= No, U= Unclear, NA= Not applicable								
Items	Willock et al(2016)	Foster et al (2017)	Alderden et al (2017)	Vocci et al (2017)	Sari & Altay (2017)	Broom et al (2017)	Curley et al (2018)	Riccioniet al (2019)
1- Was the test evaluated in a sample of subjects who were representative of those to whom the authors intended the results to be applied ?	Y	Y	Y	Y	Y	Y	Y	Y
2- Was the test performed by raters who were representative of those to whom the authors intended the results to be applied ?	Y	Y	Y	Y	Y	Y	Y	Y
3- Were raters blinded to the findings of other raters during the study ?	U	Y	NA	N	Y	U	Y	Y
4- Were raters blinded to their own prior findings of the test under evaluation ?	U	NA	NA	N	U	U	Y	U
5- Were raters blinded to the subjects' disease status or the results of the accepted reference standard for the target disorder (or variable) being evaluated ?	U	N	N	N	N	U	U	N
6- Were raters blinded to clinical information that was not intended to form part of the study design or testing procedure ?	U	U	U	U	U	U	U	U
7- Were raters blinded to additional cues that are not part of the test ?	U	U	N	U	U	U	U	U
8- Was the order of examination varied ?	U	U	U	U	Y	U	Y	Y
9- Was the stability (or theoretical stability) of the variable being measured considered when determining the suitability of the time interval among repeated measures ?	U	Y	Y	U	Y	U	Y	Y
10- Was the test applied correctly and interpreted appropriately ?	Y	Y	Y	Y	Y	U	Y	Y
11- Were appropriate statistical measures of agreement used ?	Y	Y	Y	N	Y	N	Y	Y
Y= Yes, N= No, U= Unclear, NA= Not applicable								
Items	Vocci et al (2020)	Yalçin et al (2020)	Puspitasari et al (2020)	Vocci et al (2021)	Lauderbaug et al (2021)	Luo et al (2021)	Vidal Santos et al (2022)	Verdon et al (2022)
1- Was the test evaluated in a sample of subjects who were representative of those to whom the authors intended the results to be applied ?	Y	Y	Y	Y	Y	Y	Y	Y
2- Was the test performed by raters who were representative of those to whom the authors intended the results to be applied ?	Y	Y	Y	Y	Y	Y	Y	Y
3- Were raters blinded to the findings of other raters during the study ?	U	Y	U	U	U	Y	Y	Y
4- Were raters blinded to their own prior findings of the test under evaluation ?	U	U	U	U	U	Y	N	N
5- Were raters blinded to the subjects' disease status or the results of the accepted reference standard for the target disorder (or variable) being evaluated ?	U	U	U	U	U	U	U	U
6- Were raters blinded to clinical information that was not intended to form part of the study design or testing procedure ?	U	U	U	U	U	U	U	U
7- Were raters blinded to additional cues that are not part of the test ?	U	U	U	U	U	U	U	U
8- Was the order of examination varied ?	U	Y	U	U	U	Y	Y	Y
9- Was the stability (or theoretical stability) of the variable being measured considered when determining the suitability of the time interval among repeated measures ?	U	Y	U	U	Y	N	N	U
10- Was the test applied correctly and interpreted appropriately ?	U	Y	Y	U	Y	Y	Y	Y
11- Were appropriate statistical measures of agreement used ?	N	Y	Y	N	Y	Y	Y	Y
Y= Yes, N= No, U= Unclear, NA= Not applicable								
Items	Curcio et al (2022)	Vocci et al. (2023)	Shi and Li (2023)	Curcio et al (2024)				
1- Was the test evaluated in a sample of subjects who were representative of those to whom the authors intended the results to be applied ?	Y	Y	Y	Y				
2- Was the test performed by raters who were representative of those to whom the authors intended the results to be applied ?	Y	Y	Y	Y				
3- Were raters blinded to the findings of other raters during the study ?	Y	Y	NA	Y				
4- Were raters blinded to their own prior findings of the test under evaluation ?	U	Y	N	Y				
5- Were raters blinded to the subjects' disease status or the results of the accepted reference standard for the target disorder (or variable) being evaluated ?	U	U	U	N				
6- Were raters blinded to clinical information that was not intended to form part of the study design or testing procedure ?	U	Y	U	N				
7- Were raters blinded to additional cues that are not part of the test ?	U	Y	U	Y				
8- Was the order of examination varied ?	U	N	N	Y				
9- Was the stability (or theoretical stability) of the variable being measured considered when determining the suitability of the time interval among repeated measures ?	U	Y	U	U				
10- Was the test applied correctly and interpreted appropriately ?	Y	Y	Y	Y				
11- Were appropriate statistical measures of agreement used ?	Y	Y	Y	Y				

Y= Yes, N= No, U= Unclear, NA= Not applicable

## 4 Discussion

This systematic review aimed to provide an overview of validated pressure injury risk assessment scales for pediatric populations, with a focus on their psychometric properties and clinical applicability.

### 4.1 Main findings

Ten pediatric pressure injury risk assessment scales were identified. Most originated from adaptations of adult tools and integrated pediatric-relevant factors such as continence, moisture, nutrition, and especially mobility—a factor consistently present across all tools. This observation is consistent with Kottner et al.’s 2013 systematic review, which identified mobility as a central item in all twelve scales they evaluated (Kottner et al., 2013). The link between mobility and pressure injury development is well-established (Bluestein and Javaheri, 2008, Strobel, 2001), reaffirming its critical importance in pediatric assessment tools

Our review confirms that despite an increase in the number of validation studies since Kottner et al. (Curley et al., 2018) —from three to twenty-four— many of the included studies still suffer from important methodological limitations, such as convenience sampling, inadequate reporting of psychometric data, or inappropriate statistical analyses. For example, studies by Vocci et al (Vocci et al., 2017) and Puspitasari et al (Puspitasari et al., 2020) reported Pearson correlation coefficients to evaluate reliability — an approach inappropriate for assessing inter-rater agreement.

Regarding clinical settings, most scales were developed for pediatric and neonatal intensive care units. Some tools, like the Braden Q+P, were specifically created for surgical units (Galvin and Curley, 2012). Importantly, medical devices have emerged as a key risk factor in recent literature (Tawhara and Forster, 2021), yet only four of the ten tools (Braden Q+P, Glamorgan, Pediatric Pressure Ulcer Prediction and Evaluation Tool, and the Skin Injury Risk Assessment and Prevention scale) explicitly account for this factor in their item structure.

Among the tools reviewed, the Braden QD, Glamorgan, and Braden Q+P scales appeared the most comprehensive. The Braden QD scale was the most frequently evaluated and includes specific risk factors such as exposure to medical devices and hemodynamic instability. However, other tools—such as the Glamorgan scale (designed for pediatric intensive care units) and the Braden Q+P (surgical settings)—also demonstrate strong reliability and validity within their respective contexts. Therefore, no single tool can be considered universally superior; the choice of scale should be guided by patient characteristics, clinical setting, and purpose of use.

### 4.2 Implications for clinical practice

The National and European Pressure Ulcer Advisory Panel (NPUAP and EPUAP) societes (Strobel, 2001, Black et al., 2007) recommend that risk assessment tools consider both primary (e.g., tissue perfusion, moisture, nutrition) and secondary factors (e.g., age, oxygenation, hematological parameters). Based on the evidence reviewed, we recommend selecting pressure injury risk assessment tools according to both the care setting and the child’s age. For neonates and infants in neonatal intensive care units, the Braden QD or Neonatal Braden Q scales are appropriate due to their consideration of device-related risks. In pediatric intensive care units, the Braden QD scale is recommended for all age groups given its broad scope and validation across multiple populations. In surgical and perioperative contexts, the Braden Q+P scale is most suitable as it includes factors related to positioning and operative duration. For general pediatric wards, the Braden QD, Glamorgan, and Braden Q scales are all suitable options for infants and children, as they offer acceptable reliability and item coverage; the choice should depend on clinical context and tool availability. However, existing tools still vary widely in scope, content, and psychometric robustness. A universal, pediatric-specific tool that integrates all relevant risk domains and adheres to COSMIN standards is still lacking and urgently needed (COSMIN 2024).

## 5 Strengths & limitations

This is the second systematic review on pediatric pressure injury risk assessment tools since Kottner et al. (2013) (Curley et al., 2018). Our comprehensive and language-unrestricted search across five major databases yielded a broader set of studies and tools. All included studies were assessed using standardized instruments (QUADAS-2, QAREL), and psychometric outcomes were interpreted using COSMIN guidelines to ensure consistency across the review.

However, several limitations must be noted. Many of the included studies used non-random, convenience sampling, potentially limiting the generalizability of their findings. Some lacked full psychometric data or used suboptimal statistical methods, which reduced the precision of our comparisons. Furthermore, publication bias and database coverage may have restricted the visibility of additional relevant tools or validation studies. Lastly, while we synthesized psychometric findings, we did not perform a meta-analysis due to heterogeneity in study designs and outcome measures.

## 6 Conclusions

This review highlights the critical importance of validated pressure injury risk assessment tools for pediatric populations to detect risk early and implement timely preventive measures. Although all identified tools showed acceptable inter-rater reliability and internal consistency, none demonstrated universal superiority. The Braden QD, Glamorgan, Braden Q and Braden Q+P scales appear to be the most comprehensive and aligned with current international recommendations, but their use should be tailored to clinical context and patient profile.

Given the persistent variability in design, content, and psychometric quality, the development of a new, universally applicable pediatric scale—covering all relevant risk factors and built upon COSMIN-compliant methodology—is warranted to improve care and reduce pressure injury incidence in children.

## Funding sources

No external funding.

## CRediT authorship contribution statement

**Faustine Dessi:** Writing – review & editing, Writing – original draft, Visualization, Software, Methodology, Investigation, Formal analysis, Data curation, Conceptualization. **Julien Valeille:** Writing – review & editing, Writing – original draft, Visualization, Validation, Supervision, Methodology, Conceptualization. **Pascale Beloni:** Writing – review & editing, Writing – original draft, Visualization, Validation, Supervision, Conceptualization. **Jean Toniolo:** Writing – review & editing, Writing – original draft, Visualization, Validation, Supervision, Software, Methodology, Investigation, Formal analysis, Data curation, Conceptualization.

## Declaration of competing interest

The authors declare that they have no known competing financial interests or personal relationships that could have appeared to influence the work reported in this paper.

## Appendix Supplementary materials

Image, application 1
