# Supplementary material for: Assessment tools for the risk of pressure injury in children: A systematic review
Source: Int J Nurs Stud Adv. 2025 Aug 13;9:100410. doi: 10.1016/j.ijnsa.2025.100410 (PMC12801159; doi:10.1016/j.ijnsa.2025.100410)
Supplement: Supplementary file 1 [file mmc1.docx]

| Database | Equation | Articles identified till 30 March 2024 |
| --- | --- | --- |
| PubMed | ("Pressure Ulcer"[Mesh] OR "Pressure Injury"[Mesh]) AND ("Child"[Mesh] OR "Pediatrics"[Mesh] OR "Infants"[Mesh] OR "Neonates"[Mesh]) AND ("Risk Assessment"[Mesh] OR "Scales"[Title/Abstract] OR "Evaluation"[Title/Abstract]) | 95 |
| CINALH | ((MH "Pressure Ulcer+") OR (MH "Pressure Injury")) AND ((MH "Child+" OR MH "Pediatrics+" OR MH "Infants+" OR MH "Neonates+")) AND (MH "Risk Assessment+" OR MH "Scales" OR MH "Evaluation") | 146 |
| Web of science | (TS=("Pressure Ulcer*") OR TS=("Pressure Injury")) AND (TS=("Child*") OR TS=("Pediatric*") OR TS=("Infant*") OR TS=("Neonate*")) AND (TS=("Risk Assessment*") OR TS=("Scale*") OR TS=("Evaluation*")) | 122 |
| Embase | ('pressure ulcer'/exp OR 'pressure injury') AND ('child'/exp OR 'pediatric'/exp OR 'infant'/exp OR 'neonate') AND ('risk assessment'/exp OR 'scale':ab,ti OR 'evaluation':ab,ti) | 508 |
| Google Scholar | assessment tool for the risk of pressure ulcer in child | 93 |
| Total | | 964 |

Appendix 1. Additional Table 1: Search strategy utilized for each database incorporated in the review.
